# The protective effects of human milk-derived peptides on the pancreatic islet biology

**DOI:** 10.1242/bio.049304

**Published:** 2020-08-14

**Authors:** Amitoj Singh, Ashwantha Kumar Enjapoori, Yann Gibert, Karen M. Dwyer

**Affiliations:** School of Medicine, Faculty of Health, Deakin University, 75 Pigdons Road, Waurn Ponds, Geelong, VIC 3216, Australia

**Keywords:** Type 1 diabetes, β-cell, Regeneration, Human β-casomorphin, Bovine β-casomorphin, Zebrafish, Pancreas

## Abstract

Several epidemiological studies support the protective role of breastfeeding in reducing the risk for type 1 diabetes. Human breast milk is the perfect nutrition for infants and contains many complex proteins, lipids and carbohydrates. In this study, we examined the physiological effects of human milk-derived opioid peptides, β-casomorphins (BCM), and compared them with bovine-milk-derived opioid peptides on pancreatic hormone regulation and β-cell regeneration. Exposure of wild-type zebrafish embryos to 50 µg/ml of human BCM-5 and -7 from 3 days post fertilisation until 6 days post fertilisation resulted in an increased insulin domain of expression while exposure to bovine BCM-5 and -7 significantly reduced the insulin domain of expression as analysed by whole-mount *in situ* hybridisation. These changes may be accounted for by reduced insulin expression or β-cell number and were mitigated by the µ-opioid receptor antagonist, naloxone. The effect of BCM on β-cell regeneration was assessed following ablation of β-cells in *Tg* (*ins*: CFP-NTR) zebrafish from 3 days post fertilisation to 4 days post fertilisation, followed by exposure of bovine and human BCM-5 and -7 (50 µg/ml) from 4 days post fertilisation until 7 days post fertilisation. The regenerative capacity of β-cells was not impeded following exposure to human BCM-5 and -7, whereas the capacity of β-cells to regenerate following bovine BCM-5 and -7 exposure was reduced. Our data suggest that human BCM-5 and -7 may promote β-cell development and enable the regeneration of β-cells, while the bovine-milk-derived peptides, BCM-5 and -7, play an opposite role. These data may provide some biological explanation for the protective effect of breastfeeding on the development of type 1 diabetes.

## Abstract (precis)

**Summary:** Human mild contains bioactive peptides – β-casomorphins (BCM) – that influence β-cell biology. Specifically human BCM increased the insulin domain of expression in zebrafish embryos and did not interfere with β-cell regeneration. This may have relevance for breastfeeding practices.

## INTRODUCTION

The incidence and prevalence of type 1 diabetes (T1D) has increased worldwide over the past 2–3 decades (Mathis and Vence, 2001; Katsarou et al., 2017). Although certain genetic traits are associated with T1D (Steck and Rewers, 2011; Pociot and Lernmark), the influence of environment is evidenced by the observation that migrants acquire the same risk of T1D as the populations in the new area of residence (Söderström et al., 2012; Oilinki et al., 2012). Viral infections (Filippi and von Herrath, 2008), lifestyle factors, antibiotic use and dietary factors (Katsarou et al., 2017; Oilinki et al., 2012; Butalia et al., 2016; Yeung et al., 2011) have all been implicated in the increasing incidence of T1D. Combined with permissive gut factors such as abnormal mucosal immunity, local inflammation or altered gut permeability (Chia et al., 2017), triggers operational early in life may produce autoantibodies in children with a genetic susceptibility to T1D (Kimpimaki et al., 2001).

Breast milk is the first source of nutrition for many newborns and the World Health Organization (WHO) recommends ‘mothers exclusively breastfeed infants for the child's first six months to achieve optimal growth, development and health. Thereafter, they should be given nutritious complementary foods and continue breastfeeding up to the age of two years or beyond’ (WHO and UNICEF, 2003). Lund-Blix and colleagues examined feeding practices in Norway and Denmark and showed that infants who had never been breastfed had a twofold increase in T1D, implying that breastfeeding might play a protective role in the relationship between dietary factors and T1D (Lund-Blix et al., 2017; Rewers and Ludvigsson). The mechanism underpinning these observations however are unknown.

Human milk contains biologically active substances including the β-casomorphin (BCM) peptides. Both human and bovine milk-derived β-casomorphins (hBCM and bBCM) have been reported to have opioid properties (Brantl and Teschemacher, 1983; Ziadrou et al., 1979). bBCM-5 (Tyr-Pro-Phe-Pro-Gly), bBCM-7 (Tyr-Pro-Phe-Pro-Gly-Pro-Ile), hBCM-5 (Tyr-Pro-Phe-Val-Glu) and hBCM-7 (Tyr-Pro-Phe-Val-Glu-Pro-Ile) are obtained as a result of C-terminal cleavage of the parent β-casein protein by proteolytic degradation in the gut (Teschemacher et al., 1997). These peptides are nearly identical except for the substitution of (Val-Glu) at positions 54 and 55 of the human β-casein, with (Pro-Glu) at positions 63 and 64 in the bovine β-casein (Koch et al., 1985). We hypothesised that these differences between human and bovine BCM peptide sequence would differentially affect β-cell biology and thus the aim of this study was to examine the effect of hBCM on β-cell development and regenerative capacity and compare with bBCM.

We used zebrafish to compare the effect of hBCM to bBCM on the β-cell biology as zebrafish embryos offer unique advantages for the study of diabetes. The zebrafish pancreas share its basic organization and cell types with the mammalian pancreas (Prince et al., 2017). Like the mammalian pancreas the zebrafish is composed on an exocrine and endocrine pancreas with alpha, beta, delta, epsilon and pp cells (Li et al., 2009). The zebrafish pancreas starts developing from 24 h post fertilisation (hpf), and β-cells start expressing insulin as early as 15 hpf (Kinkel and Prince, 2009). The zebrafish endocrine function of glucose homeostasis is highly conserved between zebrafish and mammals (Maddison et al., 2015). However, zebrafish offer a unique advantage for the study of diabetes and one that lost in mammals: they can regenerate after ablation or injury without the need of external stimuli (Tiso et al., 2009).

In addition, we used the *Tg* (*ins*: CFP-NTR) zebrafish embryos that permit the preferential ablation, as well as direct visualisation, of the β-cells tagged with cyan fluorescent protein (CFP) (Pisharath et al., 2007). Using these embryos, the effect of hBCM-5 and -7 and bBCM-5 and -7 on the regenerative capacity of the β-cells was determined.

## RESULTS

### Human BCMs augment the domain of expression of insulin

**Fig. 1.**
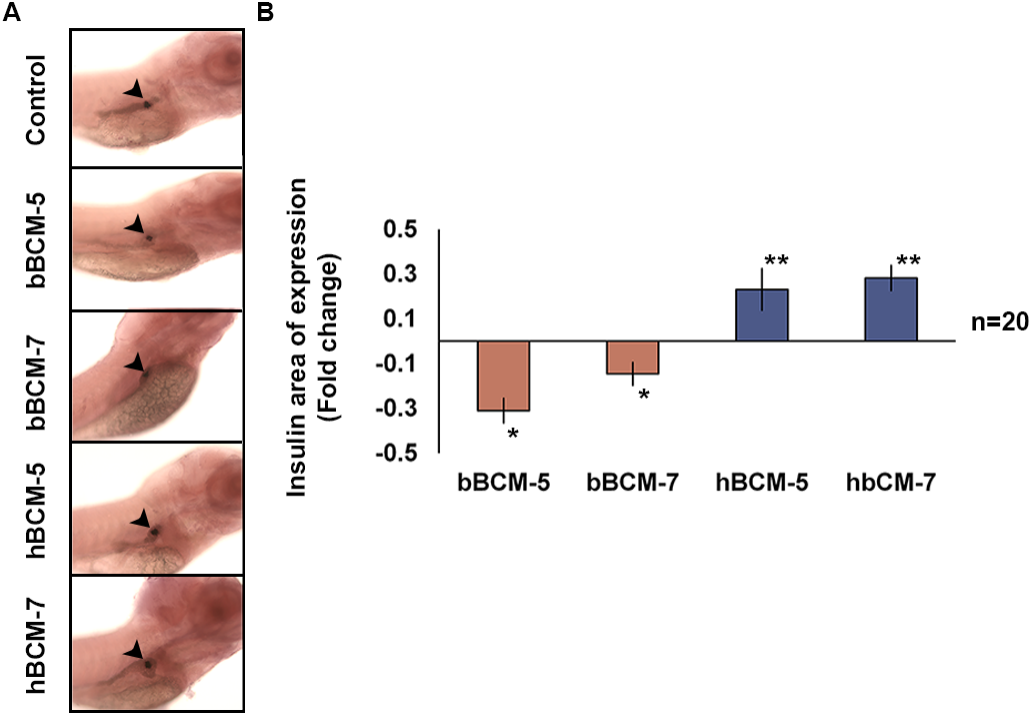
**WISH** **to analyse the effect of chronic β-casomorphin exposure on insulin domain of expression.** (A) Insulin domain of exposure (black arrowheads) in WT zebrafish embryos exposed to 50 µg/ml of bBCM-5, bBCM-7, hBCM-5 and hBCM-7 from 3–6 dpf. (B) Statistical analysis of insulin domain of expression using Student's *t*-test. Fold change (compared to untreated control zebrafish embryos) shows insulin domain of expression significantly decreased following bBCM-5 or -7 exposure and significantly increased following hBCM-5 or -7 exposure. Data represent mean±s.e.m.; *P<0.05, **P<0.005; Student's *t*-test (*n*=20).

To determine the effect of BCM on β-cell biology, wild-type (WT) embryos were exposed to bovine and human BCM-5 and -7 from 3–6 days post fertilisation (dpf) and analysed by whole-mount *in-situ* hybridisation (WISH). Exposure to bBCM-5 and -7 resulted in a significant decrease in the insulin domain of expression (*n*=20, *P*<0.05), while exposure to hBCM-5 and -7 significantly increased the insulin domain of expression (*n*=20, *P*<0.005) as compared to untreated controls (Fig. 1A,B). These data suggest that BCMs may impact the insulin domain of expression by either modulating β-cell number or function.

**Fig. 2.**
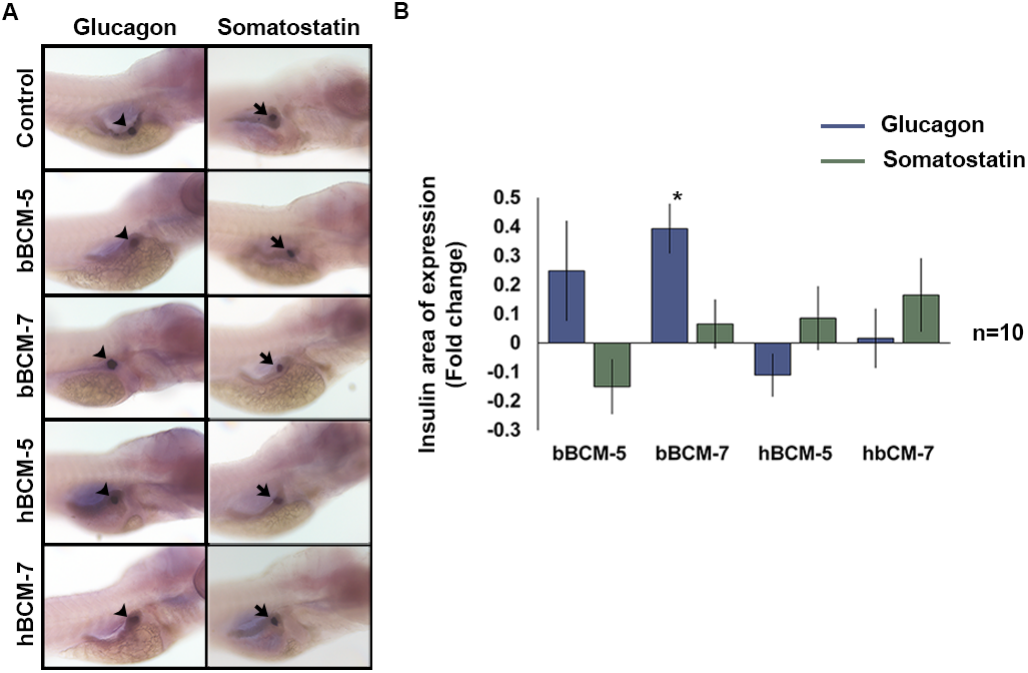
**WISH to analyse the effect of chronic β-casomorphin exposure on glucagon and somatostatin domain of expression.** (A) Glucagon (black arrowheads) and somatostatin (black arrows) domain of exposure in WT zebrafish embryos exposed to 50 µg/ml of bBCM-5, bBCM-7, hBCM-5 and hBCM-7 from 3–6 dpf. (B) Statistical analysis of glucagon and somatostatin domain of expression using Student's *t*-test. Fold change (compared to untreated control zebrafish embryos) shows glucagon domain of expression significantly increased following bBCM-7 exposure. No significant change in glucagon and somatostatin domain of exposure was detected following bBCM-5, hBCM-5 and -7 exposure. Data represent mean±s.e.m.; *P<0.05; Student's *t*-test (*n*=10).

The glucagon domain of expression was increased (*n*=10, *P*<0.05) following bBCM-7 treatment, while no change was observed with bBCM-5, hBCM-5 and -7. No significant change in the somatostatin domain of expression was observed (Fig. 2A,B) in any group. These data demonstrate that BCMs primarily affect the domain of expression of insulin suggesting that the main effect is on β-cells.

### The effect of BCMs on insulin expression is mediated via the µ-opioid receptor

**Fig. 3.**
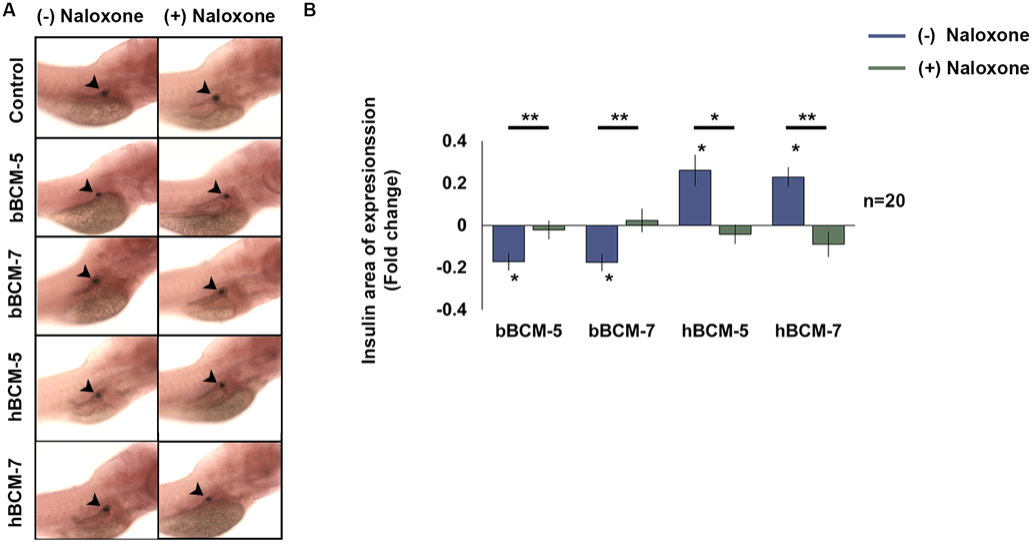
**WISH analysis of the opioid nature of the β-casomorphins.** (A) WT zebrafish embryos were pre-treated with 10 μM naloxone for 2 h to block the μ-opioid receptors and exposed to naloxone and 50 µg/ml of bBCM-5, bBCM-7, hBCM-5 and hBCM-7 every 24 h from 3–6 dpf. (Black arrowheads represent the insulin domain of expression). (B) Statistical analysis of the insulin domain of expression using Student's *t*-test. Fold change (compared to untreated control zebrafish embryos) shows insulin domain of expression significantly decreased following bBCM-5, -7 exposure and significantly increased following hBCM-5, -7 exposure. WT embryos treated with µ-opioid receptor antagonist, naloxone and bBCM-5, -7 and hBCM-5, -7 showed no changes in the insulin domain of expression compared to untreated controls. Data represent mean±s.e.m.; *P<0.05, **P<0.005; Student's *t*-test. (*n*=20).

BCMs act via the µ-opioid receptors (Koch et al., 1985; Teschemacher et al., 1997), which are located on pancreatic β-cells (de Velasco et al., 2009). The µ-opioid receptor in zebrafish shares close functional similarities with the mammalian µ-opioid receptor (de Velasco et al., 2009). WT zebrafish embryos were pre-treated with the µ-opioid receptor antagonist naloxone. The changes in the domain of expression of insulin induced by treatment with human and bovine BCM were mitigated with exposure to naloxone with levels approaching that of untreated controls (Fig. 3A and B). These data indicate that the effects of BCMs on β-cell biology are mediated via the µ-opioid receptors.

### β-cell regenerative capacity is unimpeded by human BCM-5 and -7

β-cells retain the potential to proliferate and regenerate under pathophysiological conditions including T1D (reviewed by Wu et al., 2015). To determine whether BCMs affect β-cell regeneration *Tg* (*ins*: CFP-NTR) embryos were treated with MTZ to ablate the β-cells and then exposed to BCMs.

**Fig. 4.**
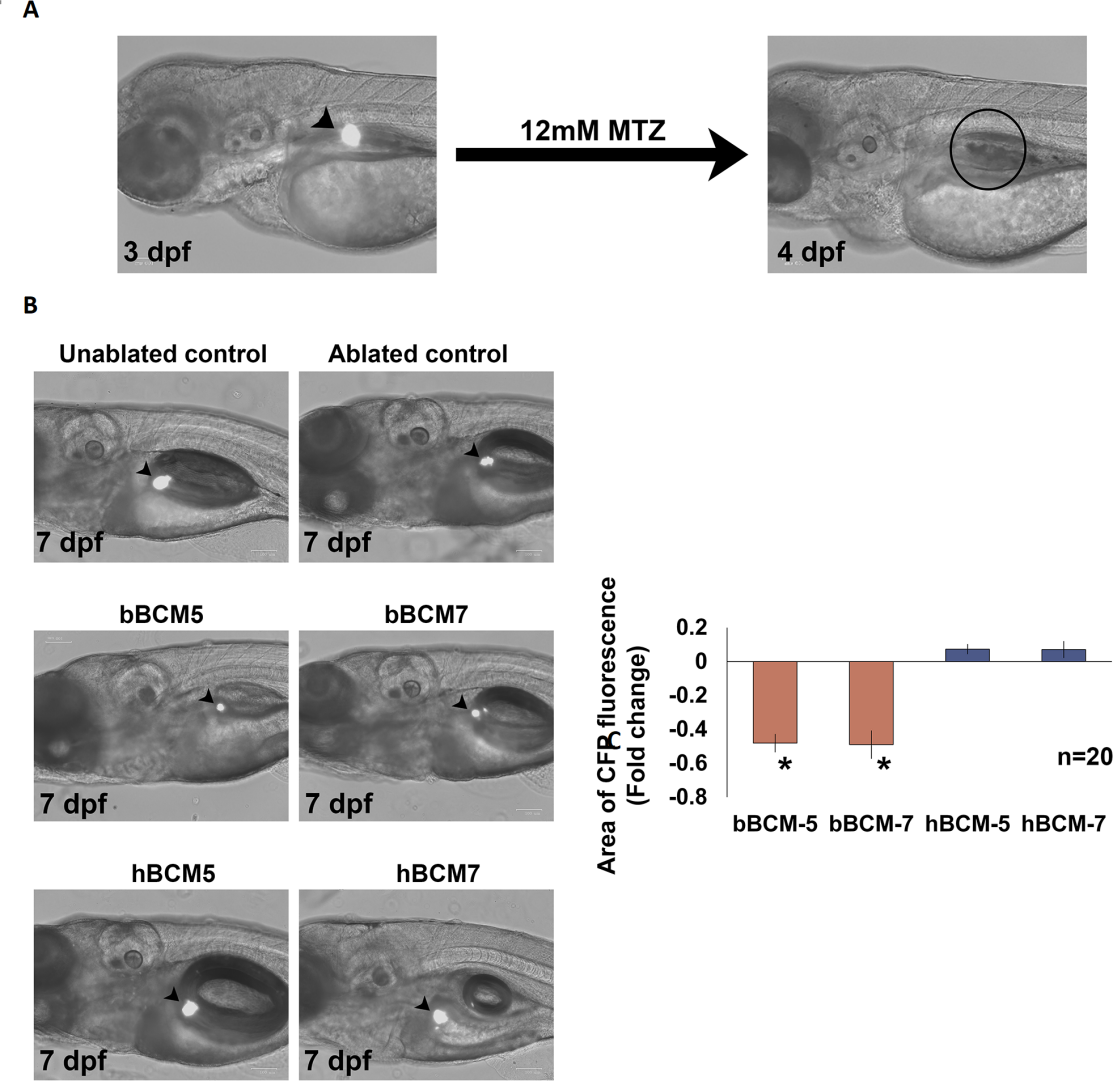
**Effect of β-casomorphins on β-cell regeneration.** (A) β-cell ablation in *Tg* (*ins*: CFP-NTR was performed using 12 mM MTZ from 3–4 dpf. No positively fluorescent cells were detected at 4 dpf indicating complete ablation (black arrowheads indicate β-cells). (B) *Tg* (*ins*: CFP-NTR) zebrafish embryos were treated with 12 mM MTZ from 3–4 dpf to ablate the pancreatic β and exposed to daily 24 h treatments of bBCM-5, bBCM-7, hBCM-5 and hBCM-7 from 4–7 dpf to analyse the effects of β-casomorphins on β-cell regeneration (black arrowheads indicate β-cells). (C) Statistical analysis using Student's *t*-test revealed a significant decrease in the area of CFP fluorescence (measured using Fiji) following treatment with bBCM-5 and bBCM-7 compared to ablated controls at 7 dpf. No changes were detected in the area of CFP fluorescence following treatment with hBCM-5 and hBCM-7 compared to ablated controls at 7 dpf. Data represent mean±s.e.m.; *P<0.0005; Student's *t*-test. (*n*=20).

Treatment with 12 mM MTZ completely ablated β-cells in *Tg* (*ins*: CFP-NTR) embryos (Fig. 4A), which had regenerated at 7 dpf (Fig. 4B). Embryos treated with bBCM-5 and -7 following ablation showed a significantly reduced area of CFP fluorescence at 7 dpf compared to control zebrafish embryos (*n*=20, *P*<0.0005) (Fig. 4B and C) indicating that the number and/or size of the β-cells was decreased. The area of fluorescence observed with *Tg* (*ins*: CFP-NTR) embryos treated with hBCM-5 and -7 at 7 dpf (*n*=20, *P*>0.1) was similar to controls (Fig. 4A and B). These data suggest that bBCM-5 and -7 negatively impacted the regenerative potential of β-cells, whereas hBCM-5 and -7 allowed β-cell regeneration to proceed.

## DISCUSSION

T1D is an autoimmune disease, triggered by an exposure to one or more environmental risk factor in a genetically susceptible individual (De Noni et al., 2009). T1D is a disease of the young suggesting that the influencing environmental triggers are operational early in life (Maahs et al., 2010). As exclusive breastfeeding is the recommended dietary source for the first 6 months of life, we sought to investigate if the human milk-derived bioactive peptides hBCM-5 and -7 may influence β-cell biology. Our data showed that the hBCM-5 and -7 promoted the domain of expression of insulin in β-cells during development, which was mediated via the µ-opioid receptor. We also demonstrated that hBCM-5 and -7 did not impede β-cell regeneration following ablation. These data are distinct to the effects of bBCM-5 and -7, which reduced the insulin domain of expression and retarded β-cell regeneration. It is possible that BCM peptides modulated insulin expression or had a direct effect on β-cell number to cause this effect and quantification of β-cell number will be required to determine the predominant effect.

It is intriguing that human and bovine BCMs induced opposing effects on the insulin domain of expression in WT embryos and yet the effects of both were neutralised with naloxone treatment. Naloxone is a competitive opioid antagonist with the highest affinity for the μ receptor but with activity at other opioid receptors. These data suggest that both forms of BCMs signal through opioid receptors on the β cell yet with divergent effects. Human islets express mRNAs for δ, and μ and NOP opioid receptors (Amisten et al., 2013). Activation of the Gi-coupled δ and μ receptors inhibits insulin release and stimulates glucagon release *in vivo* in humans (Giugliano et al., 1989) and is consistent with what was observed in response to bovine BCM, in particular bBCM-7, suggesting that these receptors may be involved in the observed effect. In contrast, human BCM-5 and -7 increased the insulin domain of expression without an effect on glucagon expression. It is possible that human BCM is signalling via alternate opioid receptors, such as NOP, in which data on the effect on islet hormone secretion is lacking (Amisten et al., 2013). It is also worthy of note that G-protein coupled receptors, including opioid receptors, undergo homo- and heterodimerisation. Receptor dimerisation can influence the potency of agonists (Wang et al., 2005) as well as downstream signalling pathways and may be operational in the effect of human BCM on β-cell biology in this instances (Rozenfeld and Devi, 2007).

Furthermore, bovine BCMs retarded β-cell regeneration, whereas those exposed to hBCMs proceeded unhindered, although were not augmented. Blocking experiments were not conducted and thus it is assumed here that bovine BCMs are signalling via δ and/or μ receptors inhibiting β-cell regeneration. In this instance, the possible alternate opioid-receptor signalling of human BCMs has no effect on regenerative capacity of β cells.

Debate around the role of breastfeeding and onset of T1D has existed for decades. Paronen and colleagues highlighted the protective effects of breastfeeding on the incidence of T1D (Paronen et al., 2000). The study concluded that infants with an increased genetic risk of developing T1D and exclusively breastfed had no IgG antibodies to insulin, while infants exposed to cows' milk develop IgG antibodies to bovine insulin (Paronen et al., 2000). Similarly, the Norwegian environmental triggers of type 1 diabetes study (MIDIA) demonstrated a protective association of breastfeeding (Lund-Blix et al., 2015), which was independent of the duration of breastfeeding (Lund-Blix et al., 2017). Several other epidemiological studies highlight the protective effect of breastfeeding in lowering the risk of T1D (Lund-Blix et al., 2017; Lucas et al., 1981; Mayer et al., 1988; Patelarou et al., 2012). However, the data are conflicting. Hakola et al. explored the associations between food diversity in infancy and the risk of islet autoimmunity or type 1 diabetes. In a sub analysis, they showed that shorter breastfeeding duration was associated with increased risk of advanced islet autoimmunity, but not type 1 diabetes, concluding that the protective effect of breastfeeding was weak (Hakola et al., 2018).

Breastfeeding has inherent physiological advantages for the infant with the composition of breastmilk changing in parallel with infant growth and nutritional needs. Breast milk provides protection for the newborn gut, stimulates the neonate’s immune system and reduces inflammation (Walker, 2010). Biologically active opioid peptides like BCMs are thought to lead to calmness and sleep in infants (Sturner and Chang, 1988), normal psychomotor development and muscle tone (Kost et al., 2009). hBCM-5 and -7 are present at elevated levels in the human colostrum (Tiruppathi et al., 1990) suggesting an important role in physiological processes in newborns (Jarmołowska et al., 2007).

Our data demonstrate a previously unknown direct effect on β cells of the bioactive peptides from human milk, hBCM-5 and -7, which may play an important role in early infant pancreatic development. Several β-cell mitogens and cellular pathways have been proposed to enhance β-cell regeneration in experimental animal models (Bouwens, 2006). Although the exact mechanisms that regulate the β-cell regeneration are poorly understood, it is widely accepted that the β-cell mass has a degree of plasticity and may fluctuate in response to physiological stress (Nir et al., 2007).

The role of cows’ milk in the incidence of T1D remains controversial. Elliot and Laugesen showed in a large epidemiological study that that incidence of T1D and ischaemic heart disease was higher in countries with a higher consumption of cows’ milk (Laugesen and Elliott, 2003). Furthermore, several claims about the association of cows’-milk proteins and the immunogenic determinants of T1D (Karjalainen et al., 1992; Cavallo et al., 1996; Vaarala et al., 1999) have generated significant public interest (Atkinson and Ellis, 1997). We recently demonstrated that genetically susceptible non-obese diabetic (NOD) mice fed a diet supplemented with A1 β-casein have an increased prevalence of diabetes compared to NOD mice fed with A2 β-casein diet in which the BCM-7 shares similarities with hBCM-7 (Chia et al. 2018). The mechanisms underpinning these observations, however, remain elusive. In this study, the insulin domain of expression in zebrafish embryos decreased following exposure to bBCM-5 and -7, which may reflect a reduction in β-cell size or number. Decreased insulin levels following exogenous opioid administration have been observed and thought to be a consequence of insulin-signalling inhibition as a result of a direct crosstalk between the µ-opioid receptor and the insulin-signalling cascade (Li et al., 2003; Singh et al., 2018).

Human breast milk is recognised as the ideal first food and the best source of nutrition for newborns. Here, we have identified a previously unknown function of human milk-derived biologically active BCM peptides in insulin regulation and β-cell mass regeneration. These data indicate that breastfeeding may be advantageous to β-cell biology.

## MATERIALS AND METHODS

### Zebrafish maintenance

All animal husbandry and experimental protocols complied with the Deakin University animal ethics committee code of practice (AWC G17-2015). Embryos were obtained from WT and *Tg* (*ins*: CFP-NTR) zebrafish adults and raised in embryonic media (E3) under standard conditions at 28.5°C with a 14 h light/10 h dark cycle, and staged according to standard procedures (Kimmel et al., 1995).

### Analysis of β-cell development following β-casomorphin exposure

Bovine and human β-casomorphin-5 and -7 were purchased from Australian Biobest Biotechnology Service and characterised by amino acid analysis (AAA) (see Table S1). WT zebrafish embryos were exposed to 50 µg/ml of bovine and human BCM-5 and -7 (in vehicle, E3) every 24 h from 3–6 dpf. At 6 dpf, WT embryos were humanely killed using 0.04% tricaine solution and fixed overnight in 4% paraformaldehyde (PFA) at 4°C. The embryos were stored in 100% methanol for at least 24 h at −20°C. WISH was performed according to established methodology (Thisse et al., 2004) using insulin, glucagon and somatostatin mRNA probes (Wyett et al., 2018). The domain of expression (positively stained area in the zebrafish embryo) was measured using Fiji software and fold change (relative to untreated control) calculated.

### Analysis of the opioid receptor-mediated effects on insulin regulation by β-casomorphins

WT zebrafish embryos were pre-treated with 10 µM naloxone (in vehicle) for 2 h at 3 dpf. Following pre-treatment, embryos were exposed to daily 24 h treatments of 50 µg/ml of bBCM-5, -7 and hBCM-5, -7 from 3–6 dpf. In addition, 10 µM naloxone was also added daily to the treatment mixture from 3–6 dpf. Control zebrafish remained in vehicle only under identical conditions. The embryos were humanely killed using 0.04% tricaine solution, fixed overnight at 4°C using 4% PFA, and stored in 100% methanol at −20°C. Insulin domain of expression following WISH was measured using Fiji software and fold change calculated.

### Analysis of the regenerative capacity of β-cells following ablation and BCM exposure

*Tg* (*ins*: CFP-NTR) zebrafish embryos were treated with 12 mM metronidazole (MTZ) in vehicle for the conditional ablation of β-cells from 3–4 dpf. Following β-cell ablation, embryos were exposed to daily 24 h treatments of 50 µg/ml of bBCM-5, -7 and hBCM-5 and -7 from 4–7 dpf. Untreated *Tg* (*ins*: CFP-NTR) (unablated control) zebrafish embryos remained in vehicle from 0–7 dpf. MTZ-treated control *Tg* (*ins*: CFP-NTR) (ablated control) zebrafish embryos were transferred to vehicle alone after β-cell ablation from 3–4 dpf and remained in vehicle until 7 dpf. The embryos were euthanised at 7 dpf using 0.04% tricaine solution and fixed overnight at 4°C using 4% PFA. Fixed embryos were rinsed three times in 1x phosphate-buffered saline with Tween^®^ 20 (PBTween) for 2 min each. Embryos were incubated in 30% and 50% glycerol/PBTween (10 min each) and stored in 70% glycerol/PBTween at 4°C. Embryos were mounted in 70% glycerol/PBTween and imaged using ZOE™ fluorescent cell imager (Bio-Rad). Statistical analysis (quantification) of the regenerated β-cells was performed by measuring the area with CFP fluorescence (positive indication of β-cells) in the zebrafish embryos using Fiji software and the fold change (compared to MTZ treated controls) calculated. Significance of the results was calculated using Student's *t*-test.

### Bovine and human BCM peptide AAA

AAA was performed by the Australian proteome analysis facility at Macquarie University, Sydney, New South Wales, Australia, to determine the composition and concentration of the synthetic, bovine and human BCM-5 and -7 peptides.

The bovine and human BCM-5 and -7 peptides with a minimum purity of 98% were obtained from the Australian Biobest Biotechnology Service, Australia.
